# Sentinel lymph node biopsy versus pelvic lymphadenectomy for early-stage cervical cancer: a retrospective institutional review

**DOI:** 10.1007/s00404-025-08134-z

**Published:** 2025-07-31

**Authors:** Tiermes Marina Martin, Cristina Celada Castro, Ariel Glickman, Nuria Carreras, Andrea Valenzuela, Pere Fusté, Adela Saco, Sergi Vidal-Sicart, Aureli Torné, Berta Díaz-Feijoo

**Affiliations:** 1 Institute Clinic of Gynecology, Obstetrics, and Neonatology, Hospital Clínic, University of Barcelona, C/Villarroel, 170, 08036 Barcelona, Spain; 2 Fundació de Recerca Clínic Barcelona-Institut d’Investigacions Biomèdiques August Pi I Sunyer (FRCB-IDIBAPS), Barcelona, Spain; 3 Department of Pathology, Hospital Clínic, University of Barcelona, Barcelona, Spain; 4 Department of Nuclear Medicine, Hospital Clínic, University of Barcelona, Barcelona, Spain; 5 Facultat de Medicina i Ciències de la Salut, Universitat de Barcelona (UB), 08036 Barcelona, Spain

**Keywords:** Cervical cancer, Lymphadenectomy, Sentinel lymph node, Surgery

## Abstract

**Objective:**

To evaluate the oncologic and survival outcomes in patients diagnosed with early-stage cervical cancer who underwent both sentinel lymph node (SLN) and pelvic lymphadenectomy (PLD) compared with those who underwent SLN alone at primary surgery.

**Methods:**

From 2001 to 2022, women who underwent SLN biopsy for nodal staging were recruited. The group of women who underwent SLN biopsy and PLD (SLN + PLD group) was compared with the group who underwent SLN mapping alone (SLN group).

**Results:**

210 patients were evaluated (98 and 112 in each group). The overall SLN detection rate was 97.6%. Lymph node involvement was detected in 23 patients (11%), and the rate of positive SLN increased from 6.2 to 11% after final pathological examination. At a median follow-up of 80 months, the recurrence and mortality rates were 6.2 and 2.4%, respectively. The 3-year progression-free survival (PFS) rate was 93.7 and 97.2%, and the overall survival (OS) rate was 98.9 and 99.0% in the SLN + PLD and SLN group, respectively. There were no significant differences in the Kaplan–Meier PFS (*p* = 0.471; HR 0.66; 95% CI 0.22–2.04) and OS (*p* = 0.228; HR 0.28; 95% CI 0.03–2.53) curves between the groups.

**Conclusion:**

Pending further confirmation from prospective trials, SLN biopsy appears to be an effective method of nodal assessment in early-stage cervical cancer. This technique does not appear to increase the risk of recurrence compared with complete PLD in selected patients and may offer a viable, less invasive alternative for accurate nodal staging.

**Supplementary Information:**

The online version contains supplementary material available at 10.1007/s00404-025-08134-z.

## Introduction

Lymph node involvement is one of the most important prognostic factors in cervical cancer [1]. Nodal staging is required in order to identify these high-risk patients who may benefit from adjuvant complementary treatment [2] and has recently been included in the latest update of the International Federation of Gynecology and Obstetrics (FIGO) classification in 2018 [3].

The application of the sentinel lymph node (SLN) technique aims to accurately assess lymph node metastases involvement while reducing the morbidity associated with radical surgery and maintaining its oncologic prognosis [4–7]. Based on this evidence, international guidelines now consider SLN biopsy as a viable alternative to PLD in early-stage cervical cancer [8, 9]. However, prospective evidence on long-term oncological safety remains limited and the results of the ongoing prospective trials are expected to provide further insight [10–12].

The objective of this study is to evaluate the oncologic outcomes in early-stage cervical cancer by comparing two groups of patients over the past 20 years: a historical cohort that underwent both selective SLN biopsy and PLD and another cohort that underwent SLN biopsy alone.

## Material and methods

### Study population

We conducted a retrospective review of historical cohorts at the Hospital Clinic in Barcelona, Spain, including women diagnosed with early-stage cervical cancer (stages IA1-IIA1 according to the FIGO 2018 classification) and no suspicious nodes on preoperative imaging from May 2001 to December 2022. All participants underwent surgery including SLN assessment. We excluded patients with suspected lymph node metastasis at preoperative imaging and patients who did not have lymph node staging or who underwent systematic PLD without SLN. Surgery was performed by specialist gynecologic oncology surgeons. The project was approved by the institutional review board of Hospital Clínic Barcelona (HCB/2023/1203).

### SLN mapping procedures and surgery

**Fig. 1:**
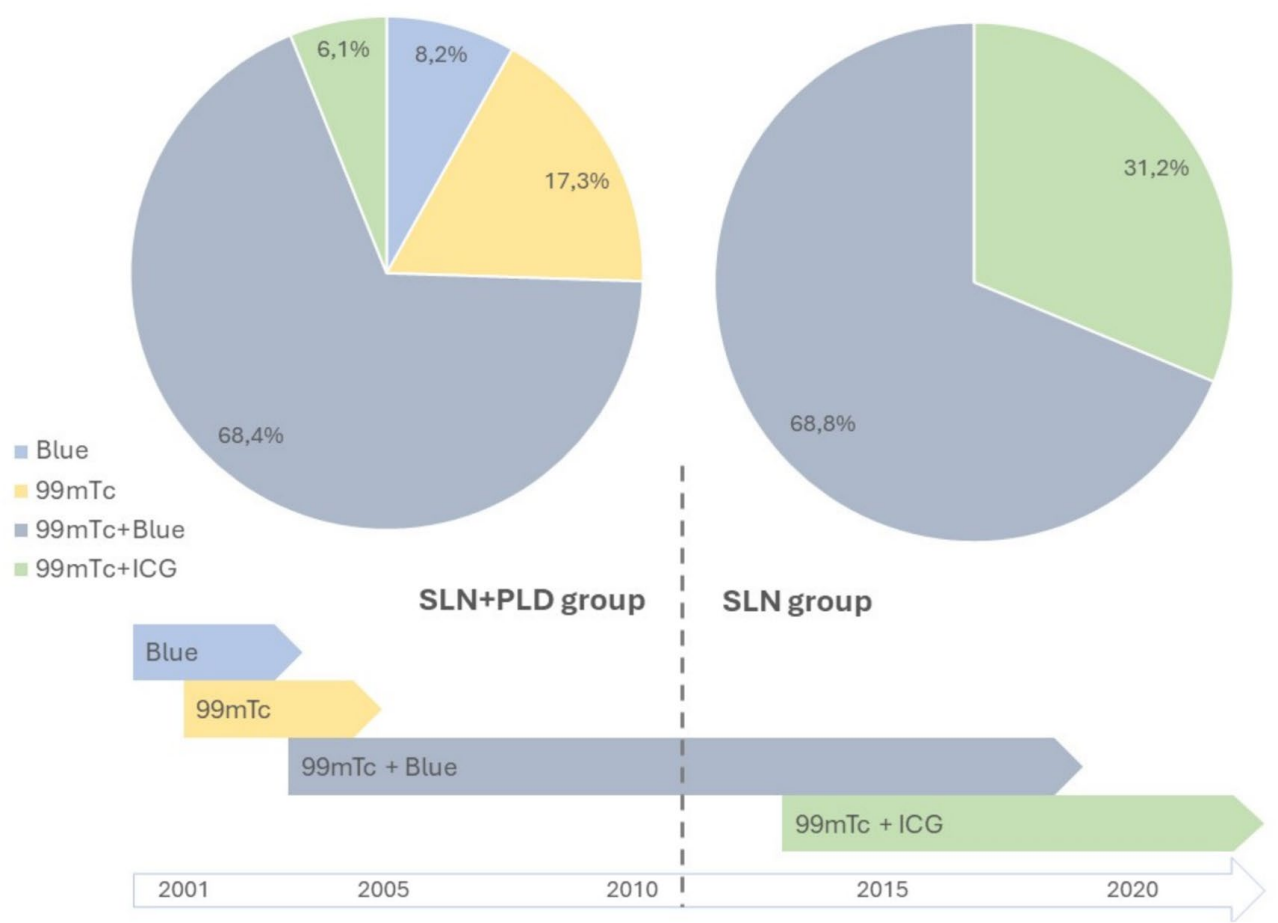
Distribution and temporal evolution of the use of dyes and radiotracers for sentinel lymph node detection between groups over time

Different tracers have been used over the years, including 99mTc-nanocolloid radiotracer (99mTc), blue dye, fluorescent dye indocyanine green (ICG) and hybrid tracer (99mTc-nanocolloid-ICG) [13]. From 2001 to 2017, the SLN mapping procedure was performed with 99mTc plus blue dye, blue dye alone, or 99mTc alone. In 2014, fluorescence detection with ICG was introduced in our department, and the hybrid tracer that is currently used was gradually incorporated [13, 14] (Fig. 1). The tracer was injected according to current guidelines. Preoperative lymphoscintigraphy and a SPECT/CT scan were also performed. Intraoperative identification of SLNs was performed using lymphatic mapping with blue dye, using a near-infrared (NIR) fluorescence imaging system (Storz Full HD D-Light P ICG) or by a laparoscopic gamma probe (Navigator GPS; RMD Instruments).

The procedure was aborted in cases of metastatic suspicious node or SLN and systematic para-aortic lymphadenectomy was performed instead. From 2001 to 2011, while our group was validating the SLN procedure in early-stage cervical cancer, patients also underwent systematic bilateral PLD after SLN dissection (SLN + PLD group) [15, 16]. From 2012 onwards, only SLN was performed (SLN group). If no SLNs were found on one or both sides of the pelvis, a systematic PLD was performed for intraoperative evaluation. If SLNs were found to be disease-free by frozen section pathological examination (FSE), radical trachelectomy or hysterectomy (laparoscopic, laparotomic or vaginal assisted) was performed.

### Histopathology

Frozen section pathologic examination at one level by hematoxylin–eosin (H&E) staining was performed in all cases. Subsequently, the SLN was sent for standardized pathologic evaluation including ultrastaging protocol. All SLNs were fixed in 10% buffered formalin, sectioned at 2 mm intervals (transverse to the major axis) and embedded in paraffin. Two consecutive sections (4 μm thick) were cut. The first section was stained with H&E; if no metastatic disease was identified, the second section was examined by immunohistochemistry (IHC) with anti-cytokeratin AE1/AE3 antibodies. From 2018 onwards, 1 H&E and 1 IHC slides were obtained from each paraffin block at 5 levels, cut at 250 μm intervals, until no lymph node tissue remained. Metastatic lymph node disease was categorized according to the American Joint Committee on Cancer definitions [17]: isolated tumor cells (ITCs) were defined as <0.2 mm, micrometastases as between 0.2 and 2 mm, and macrometastases as >2 mm.

### Adjuvant treatment and follow-up

For patients presenting a combination of risk factors at final pathology, adjuvant treatment planning was conducted at a multi-disciplinary tumor board meeting in accordance with current clinical guidelines. Follow-up included physical exams and imaging for recurrence detection.

### Data and statistical analysis

Descriptive statistics were calculated for all variables of interest. Data were reported as mean (SD) or median (ranges) for quantitative variables and as numbers (percentages) for categorical variables. For the univariate analysis, categorical variables were compared using Pearson’s *χ*^2^ test or Fisher’s exact test, and continuous variables were compared using Student’s t-test.

To evaluate the success of lymphatic mapping, sensitivity, negative predictive value, and false-negative rates were calculated per patient. A true-negative SLN was defined as SLN free of disease at both FSE and final pathologic examination. A false-negative SLN was defined as SLN considered initially as free of disease by FSE but finally metastatic after definitive pathologic examination. Survival rates were analyzed using the Kaplan–Meier method, and differences between two groups were analyzed with the log-rank test. All statistical tests were two-tailed, and the significance threshold was set at the *p* < 0.05 level. All analyses were performed with Stata version 13.1 (StataCorp. 2013. Stata statistical software: Release 13. College Station, TX).

## Results

A total of 210 patients with early-stage cervical cancer were included in the study. From 2001 to 2011, 98 patients underwent SLN mapping and PLD (SLN + PLD group) and from January 2012 onwards, 112 patients underwent SLN mapping alone (SNL group). There were no significant differences in median age or histological subtype between the groups. Compared to the SLN group, the SLN + PLD group had a higher proportion of patients with tumors larger than 2 cm (38.7 vs 28.5%) and vaginal involvement (3.1 vs 0%). The SLN + PLD group also had a higher proportion of patients who underwent radical hysterectomy (75.5 vs 66.1%). Most of the patients (65.2%) underwent laparoscopic assisted radical vaginal hysterectomy (LARVH): 74 (75.5%) in the SLN + PLD group and 63 (56.3%) in the SLN group (Table 1).

**Table 1:** Clinical-pathological and surgical features

Characteristics	SLN + PLD group *N* = 98	SLN group *N* = 112
Age at diagnosis (years), mean (SD)	45.4 (13.1)	43.3 (10.7)
FIGO stage (2018) at diagnosis, *n* (%)		
IA1/1A2	11 (11.2)	32 (28.6)
IB1	38 (38.8)	42 (37.5)
IB2	45 (45.9)	35 (31.25)
IB3	1 (1)	3 (2.7)
IIA1	3 (3.1)	0
Tumors ≤2 cm, *n* (%)	60 (61.2)	80 (71.4)
Histological type, *n* (%)		
Squamous	66 (67.3)	75 (67)
Adenocarcinoma	30 (30.6)	35 (31)
Others	2 (2.1)	2 (2)
Type of surgery, *n* (%)		
Radical hysterectomy	74 (75.5)	74 (66.1)
Radical trachelectomy	15 (15.3)	36 (32.1)
Radical surgery not completed	9 (9.2)	2 (1.8)
Surgical approach, *n* (%)		
LARVH (Coelio-Schauta)	74 (75.5)	63 (56.3)
LPS	9 (9.2)	10 (8.9)
LPT	0	3 (2.7)
Vaginal	15 (15.3)	36 (32.1)
Tracer, *n* (%)		
Blue dye	8 (8.2)	0
99mTc	17 (17.3)	0
Blue dye + 99mTc	67 (68.4)	35 (31.2)
Hybrid tracer (ICG + 99mTc)	6 (6.1)	77 (68.8)

*ICG* indocyanine green, *LARVH* laparoscopically assisted radical vaginal hysterectomy, *LPS* laparoscopy, *LPT* laparotomy, *PLD* pelvic lymphadenectomy, *RT* radiotherapy, *SLN* sentinel lymph node, *Tc* technetium

### Detection rate of SLN mapping

At least one SLN was detected in 205 women (97.6% overall detection rate, 94.9 and 100% in the SLN + PLD and SLN groups respectively) using one of the aforementioned methods (Fig. 1). Bilateral surgical SLN detection was successful in 56.1% of the SLN + PLD group and in 93.8% of the SLN group. Seven patients underwent complete unilateral pelvic node dissection on the side where no SLN was found in the SLN group. The median number of SLNs identified per patient was 3 (range 0–8) in the SLN + PLD group and 4 (range 1–8) in the SLN group. In women who underwent lymphadenectomy, the median number of lymph nodes identified was 18.6 (range 4–45) (Table 2).

**Table 2:** Surgical results

Characteristics	SLN + PLD group *N* = 98	SLN group *N* = 112
SLN performed, *n* (%)	98 (100)	112 (100)
Bilateral pelvic lymphadenectomy, *n* (%)	98 (100)	7 (6.25)
Pelvic and para-aortic lymphadenectomy, *n* (%)	11 (10.8)	4 (3.6)
Successful mapping of SLN, *n* (%)	93 (94.9)	112 (100)
Bilateral mapping, *n* (%)	55 (56.1)	105 (93.8)
Para-aortic SLN, *n* (%)	0	0
Number of SLN removed, median (range)	3 (0–8)	4 (1–8)
Number of total LN removed, median (range)	18.6 (7–45)	–
Positive SLN, *n* (%)	13 (13.3)	10 (8.9)
Positive non-SLN, *n* (%)	4 (4.1)	2 (1.8)

*LN* lymph nodes, *PLD* pelvic lymphadenectomy, *SLN* sentinel lymph node

### Incidence of SLN metastases

Lymph node involvement was detected in 23 patients (11%), 13 patients (13.3%) in the SLN + PLD group and 10 patients (8.9%) in the SLN group. Most patients (76.2%) had tumors greater than 2 cm. Intraoperative frozen section analysis diagnosed metastatic disease in 13 patients, and definitive pathological assessment detected low-volume metastatic disease in a further 10 cases, increasing the proportion of patients with positive SLNs from 6.2 to 11%. Of the 748 SLNs, metastatic involvement was diagnosed in 32 nodes. Intraoperative FSE identified 7 SLNs with macrometastases, 10 SLNs with micrometastases, while no ITCs were identified. Final pathological examination and ultrastaging of negative SLNs identified an additional 8 SLNs with micrometastases and 7 SLNs with ITCs, respectively. When considering only macrometastases and micrometastases, definitive pathological study significantly improved nodal staging by identifying an additional 6 patients with positive SLNs, representing an increase of 2.8%. In addition, metastatic disease was detected in 6 non-sentinel lymph nodes. Details of the comparative analysis between the intraoperative FSE and definitive histological examination of the SLN are shown in Table 3 and Supplementary Table S1.

**Table 3:** Metastatic SLN involvement and diagnostic evaluation of the SLN technique per patient and group

	SLN + PLD group *N* = 98		SLN group *N* = 112		Total *N* = 210	
	FSE	Definitive pathologic examination	FSE	Definitive pathologic examination	FSE	Definitive pathologic examination
Positive lymph node, *n* (%)						
All types	10 (10.2)	13 (13.3)	3 (2.7)	10 (8.9)	13 (6.2)	23 (11)
Positive lymph node, *n* (%)						
Mic and Mac (excluding ITCs)	10 (10.2)	13 (13.3)	3 (2.7)	6 (5.4)	13 (6.2)	19 (9)
Type SLN involvement, *n* (%)						
Macrometastases	5 (5.1)	5 (5.1)	2 (1.8)	2 (1.8)	7 (3.3)	7 (3.3)
Macrometastases	5 (5.1)	8 (8.2)	1 (0.9)	4 (3.6)	6 (2.9)	12 (5.7)
ITC	0	0	0	4 (3.6)	0	4 (1.9)
No	88 (89.8)	85 (86.7)	109 (97.3)	102 (91.1)	197 (93.8)	187 (89)

*FSE* frozen section examination, *ITC* isolated tumor cell, *Mac* macrometastases, *Mic* micrometastases, *SLN* sentinel lymph node

Frozen section examination of the SLN had a sensitivity of 56.5%, a negative predictive value of 94.9% with a false negative (FN) rate of 43.5% when compared to the definitive pathological study. The area under the curve (AUC) for FSE was 78.3% (95% CI 67.9–88.6%). When excluding ITCs, FSE achieved a sensitivity of 68.4%, a negative predictive value of 97% and a proportion of FN of 31.6%. The corresponding AUC also increased to 84.2% (95% CI 73.5–94.9%).

### Surgical results and complications

Surgical or immediate postoperative complications of any grade occurred in 21 women (10%) (11 and 10 patients in each group, respectively). Of these, 10 women (47.6%) had complications grade IIIb or worse according to the Clavien–Dindo classification. Intraoperative complications were identified in 5 patients. Urologic complications (cystostomy, ureteral injury, and bladder dysfunction) were the complications most commonly reported in 5.1 and 3.6% of the patients in each group, respectively. In addition, 15 patients had some type of complication within the first 30 days after surgery, most of them urological complications, including five patients with ureteral/vesical fistula requiring surgical intervention for repair in the SLN + PLD group. There were no significant differences in intra-operative or short-term morbidity (*p* = 0.58) between the two groups. However, in terms of mid- and long-term morbidity, the SLN + PLD group had a higher incidence of lymphedema (9.2 vs 1.8%) (*p* = 0.016). Details of intra- and post-operative complications are shown in Table 4.

**Table 4:** Follow-up

Characteristics	SLN + PLD group *N* = 98	SLN group *N* = 112	*P* value
Intraoperative or immediate postoperative complications, *n* (%)			0.580
Bowel injury	1 (1)	0	
Urological injury	5 (5.1)	4 (3.6)	
Vascular injury	1 (1)	0 (0)	
Nerve injury	0	0	
Infections	3 (3.1)	3 (2.7)	
Others	1 (1)	3 (2.7)	
Medium or long-term complications, *n* (%)			0.075
Lymphedema	9 (9.2)	2 (1.8)	0.016
Symptomatic lymphocele/lymphocystis	1 (1)	0	
Sensitive/motor symptoms	1 (1)	0	
Fistula	0	0	
Urological dysfunction	0	2 (1.8)	
Other: RT related complications	3 (3.1)	2 (1.8)	
Adjuvant treatment, *n* (%)			0.045
No	58 (59.2)	81 (72.3)	
RT (ERT/BT)	32 (32.7)	22 (19.6)	
RT + CT	8 (8.2)	9 (8)	
Recurrences, *n* (%)			0.267
Total	8 (7.8)	5 (4.5)	
Vaginal	2 (2)	3 (2.7)	
Nodal	4 (4.1)	1 (0.9)	
Pelvic	1 (1)	0	
Distant metastasis	1 (1)	1 (0.9)	

*BT* brachytherapy, *ERT* external radiotherapy, *PLD* pelvic lymphadenectomy, *QT* chemotherapy, *RT* radiotherapy, *SLN* sentinel lymph node

### Follow-up data and survival outcome

Seventy-one patients (33.6%) received adjuvant treatment for adverse risk factors: 40.8% in the SLN + PLD group versus 27.7% in the SLN group (*p* = 0.045). A higher proportion of patients in the SLN + PLD group received adjuvant radiotherapy compared to the SLN group (40.8 vs 27.7%; *p* = 0.045).

With a median follow-up period of 80 months (range 3–275), recurrence was observed in 13 women (6.2%): 8 patients in the SLN + PLD group and 5 patients in the SLN group. The median time to recurrence was 28.1 months in the SLN + PLD group and 25.6 months in the SLN group, with no significant difference between the groups (*p* = 0.189). Most recurrences (69.2%) occurred in women with tumors ≥2 cm. Detailed data on adjuvant therapy and recurrence are shown in Table 4.

**Fig. 2:**
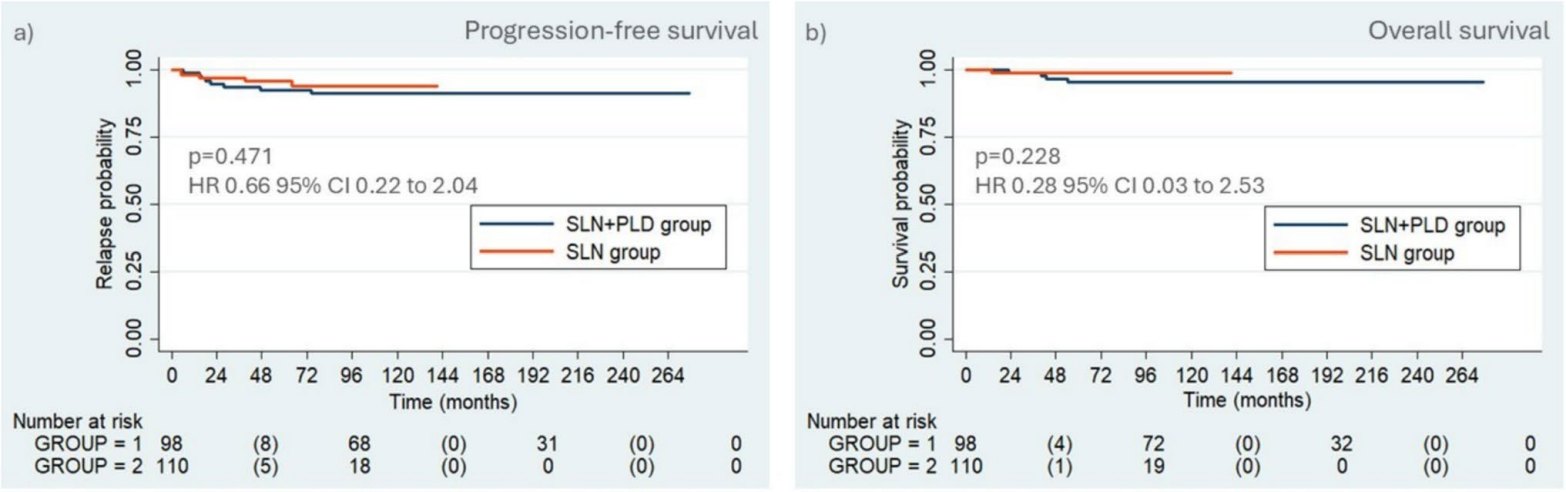
Kaplan–Meier curves for progression-free survival (PFS) (**a**) and overall survival (OS) (**b**) between SLN + PLD and SLN groups

Five patients died of cervical cancer, which represents a mortality rate of 2.4%: four in the SLN + PLD group with a median survival time of 41.5 months (range 23–54) and one in the SLN group with a median survival of 14 months. In addition, another 11 patients in the SLN + PLD group died of other diseases or neoplasia without any sign of cervical cancer recurrence at the time of death. The 3-year progression-free survival (PFS) rate was 93.7% (95% CI 86.4–97.1%) in the SLN + PLD group and 97.2% (95% CI 91.4–99.1%) in the SLN group. Overall survival (OS) rate was 98.9% (95% CI 92.7–99.9%) and 99.0% (95% CI 92.9–99.9%) in the SLN + PLD and SLN groups, respectively. There were no significant differences in the Kaplan–Meier PFS (*p* = 0.471; HR 0.66; 95% CI 0.22–2.04) and OS (*p* = 0.228; HR 0.28; 95% CI 0.03–2.53) curves between the two groups. The PFS and OS rates are shown in Fig. 2.

## Discussion

SLN dissection is increasingly recognized as a valuable method for nodal assessment in patients diagnosed with early-stage cervical cancer. This study of over 200 patients supports the oncologic safety of the technique. It shows comparable oncological outcomes between patients who underwent complete pelvic lymphadenectomy and those who underwent selective SLN biopsy alone.

Our results show no statistical differences in PFS and OS rates in both arms of our study (*p* = 0.471 and *p* = 0.228). This suggests that omitting PLD does not increase the risk of recurrence in selected patients with early cervical cancer in line with data from previous studies [6, 18–20]. This aligns also with the results of the ETERNITY project, recently published, which evaluated nodal assessment methods in a cohort of patients undergoing fertility-sparing treatment [21]. The ETERNITY project found similar disease-free and overall survival outcomes between patients undergoing sentinel node mapping alone, sentinel node mapping with back-up PLD, and PLD. Although international guidelines suggest and support it as an alternative to PLD based on all this evidence, SLN biopsy alone is not yet considered the gold standard for SLN assessment, due to the lack of prospective evidence. Three clinical trials are currently underway to reliably determine the prognostic impact of SLN application in early-stage cervical cancer: the SENTIX [10], the PHENIX [11] and the SENTICOL III [12] trials. These studies aim to confirm the reproducibility and oncologic safety of the technique for definitive implementation in clinical practice.

Related to intraoperative complications and short-term morbidity, our results show more intestinal, urological, and vascular complications in the SLN + PLD, while the SLN group had more hemorrhagic complications, but there was no significant difference in intraoperative complications or short-term morbidity between the two groups (*p* = 0.58). For long-term complications, we found a higher rate of lymphedema, symptomatic lymphocele/lymphocyst formation, and sensitive/motor symptoms in the SLN + PLD group bordering on statistical significance (*p* = 0.075), at the expense of a significant difference in the percentage of patients who developed clinical lymphedema in this group (*p* = 0.016). However, these differences may also be influenced by a higher percentage of patients receiving adjuvant radiotherapy in this group. Paralleling our findings, Lennox et al. [18], comparing two groups similar to ours, also describe no significant difference in intra-operative complications or short-term morbidity but a higher median intraoperative blood loss, median operative time, blood transfusion, median length of postoperative stay, and postoperative infection. Furthermore, a recent meta-analysis, which included results from the SENTICOL-2 clinical trial and three additional studies, showed that lymphedema was significantly less common in the SLN group compared to the PLD group (odds ratio: 0.12) [20] and the previous Bogani study in which morbidity was significantly lower in the SLN group compared to those undergoing more extensive procedures [21]. This highlights the potential benefits of the SLN approach in reducing long-term morbidities associated with radical surgery.

The excellent accuracy of SLN in detecting nodal involvement has also been demonstrated with very high overall detection rates (>90%) similar to those described in the literature [20, 22–24]. Although the bilateral detection rates were modest, they improved significantly over time and have been refined to achieve higher bilateral detection rates of up to 97% as described in recent articles [6, 20, 24, 25]. Several factors have contributed to this improvement: first, the learning curve associated with efficient cervical injection, intraoperative detection, and precise node sampling [26, 27] as demonstrated by higher detection rates in the SENTICOL II cohort (83.5%) compared to the SENTICOL I cohort (75%) (37). Second, a better understanding of lymphatic involvement and drainage pathways in cervical cancer, which has shown that although most lymph nodes are located in the pelvic area, positive nodes have also been identified in other regions [26]; this has led to a better understanding of the drainage of these tumors and therefore to a greater efforts to detect sentinel nodes in the different drainage areas. Finally, the choice of the best tracer. In the early years, the combination of the radiocolloid (99mTc) and blue dye was used and validated in our center [14]. However, the introduction of indocyanine green in the last decade has marked a significant evolution in tracer technology, showing higher or similar bilateral SLN detection rates [20, 27]. At our center, we have further improved detection capabilities by developing and using a hybrid tracer which combines the advantages of radio- and fluorescence-guided surgical techniques, thereby improving bilateral detection rates in line with published data [13].

Definitive pathological assessment, particularly ultrastaging, significantly improves the detection of LN metastases, detecting up to 20–43% more node-positive patients than with conventional frozen section [18, 26, 28–31]. Intraoperative analysis detects lymph node involvement in approximately 50–60% of patients with positive lymph nodes, effectively identifying the majority of MAC [28, 32]. Our study found a 5% increase in the detection rate of positive SLN, confirming the variable diagnostic accuracy of frozen section with a significant number of false negatives (43.5%) [33–36]. Furthermore, there has recently been evidence that the detection rate of positive SLNs correlates with the intensity of ultrastaging, with more than 90% of N1s detected using a standardized ultrastaging protocol with examination of four levels of paraffin blocks [31]. In our study, all ITC and most of the small metastases were diagnosed after ultrastaging implantation, supporting the need for a comprehensive and systematic staging study of SLN. However, the prognostic significance of low-volume metastases, especially for ITCs, remains unclear [19]. Cibula observed that the presence of micrometastases significantly reduces OS, equivalent to patients with macrometastases [30], and a recent meta-analysis confirms the negative prognostic impact of micrometastases on both DFS and OS, advocating for treatment protocols similar to those used for macrometastases [22]. No prognostic significance was found for ITC, but it should be contemplated along with other risk factors when considering adjuvant treatment [19, 30, 37]. However, the available evidence showing that MIC is a significant negative prognostic factor comes only from retrospective studies. Two ongoing European prospective trials involving more than 1000 patients (SENTIX and SENTICOL III) will provide more certainty about the significance of this low-volume nodal involvement [10, 12]; however, the prognostic implication of ITCs is likely to be more challenging to ascertain.

The present study represents one of the largest single-center analyses of recurrence outcomes in cervical cancer patients who underwent SLN biopsy, involving over 200 patients treated over a 20-year period by a team experienced in both the surgical treatment of cervical cancer and the application of SLN techniques to other tumors. However, there are several limitations to be considered. As a retrospective and observational study, it is subject to the inherent biases associated with comparing a contemporary cohort with a historical cohort. These groups differed in the protocols and guidelines followed over the years, as well as in the tracers used. In addition, there is considerable baseline heterogeneity between the groups: the SNL group, which was considered to have a better prognosis, had a significantly higher proportion of patients with microscopic tumors and a lower proportion of patients with tumors larger than 2 cm. This difference also influenced the administration of adjuvant treatment between the two groups. Such differences reflect the evolution of patient management over time in a tertiary center specializing in the treatment of gynecological malignancies.

## Conclusion

SLN biopsy shows high accuracy in detecting nodal involvement and does not appear to increase the risk of recurrence according to our findings. This suggests that it could be considered as a viable alternative to total pelvic lymphadenectomy. However, although promising, the long-term safety and efficacy of this technique need to be confirmed by prospective randomized trials.

## Supplementary Information

Below is the link to the electronic supplementary material.

Supplementary file 1 (DOCX 20 KB)

## Data Availability

No datasets were generated or analyzed during the current study.
