# Supplementary material for: Sentinel lymph node biopsy versus pelvic lymphadenectomy for early-stage cervical cancer: a retrospective institutional review
Source: Arch Gynecol Obstet. 2025 Jul 31;312(4):1327–35. doi: 10.1007/s00404-025-08134-z (PMC12414009; doi:10.1007/s00404-025-08134-z)
Supplement: Supplementary file 1 — (DOCX 20 KB) [file 404_2025_8134_MOESM1_ESM.docx]

**Table S1.** Results of positive LN per patient and group according to frozen section versus definitive pathologic examination.

| **SLN+PDL group** | | | | | | **SLN group** | | | | | |
| --- | --- | --- | --- | --- | --- | --- | --- | --- | --- | --- | --- |
| **Pat nº** | **Total**  **SLN** | **Total**  **(+) SLN FSE** | **Total**  **(+) SLN Ultrastaging** | **Total**  **(+) SLN definitive histological study** | **Total**  **(+) LN definitive histological study** | **Pat nº** | **Total**  **SLN** | **Total**  **(+) SLN FSE** | **Total**  **(+) SLN Ultrastaging** | **Total**  **(+) SLN definitive histological study** | **Total**  **(+) LN definitive histological study** |
| 1 | 1 | 1 (macro) | 0 | 1 | 1 | 14 | 8 | 0 | 1 (ITC) | 1 | 1 |
| 2 | 3 | 0 | 2 (micro) | 2 | 2 | 15 | 6 | 0 | 1 (micro) | 1 | 1 |
| 3 | 2 | 0 | 2 (micro) | 2 | 3 | 16 | 4 | 2 (micro) | 1 (ITC) | 3 | 3 |
| 4 | 5 | 1 (macro) | 0 | 1 | 4 | 17 | 1 | 0 | 1 (ITC) | 1 | 1 |
| 5 | 4 | 1 (macro) | 0 | 1 | 2 | 18 | 3 | 0 | 1 (micro) | 1 | 3 |
| 6 | 2 | 1 (macro) | 0 | 1 | 2 | 19 | 1 | 1 (macro) | 0 | 1 | 2 |
| 7 | 3 | 0 | 1 (micro) | 1 | 1 | 20 | 5 | 1 (macro) | 0 | 1 | 2 |
| 8 | 1 | 1 (macro) | 0 | 1 | 1 | 21 | 6 | 0 | 2 (ITC) | 2 | 2 |
| 9 | 3 | 1 (micro) | 0 | 1 | 1 | 22 | 2 | 0 | 1 (ITC) | 1 | 1 |
| 10 | 4 | 1 (micro) | 0 | 1 | 1 | 23 | 4 | 0 | 1 (micro) | 1 | 1 |
| 11 | 5 | 3 (micro) | 0 | 3 | 3 |  |  |  |  |  |  |
| 12 | 2 | 1 (micro) | 0 | 1 | 1 |  |  |  |  |  |  |
| 13 | 5 | 2 (micro) | 1 (ITC) | 3 | 3 |  |  |  |  |  |  |
| SLN |  | 13 | 6 | 19 | 25 |  |  | 4 | 9 | 13 | 17 |
| Pat |  | 10 | 3 | 13 | 13 |  |  | 3 | 7 | 10 | 10 |

FSE, frozen section examination; ITC, isolated tumor cells; LN, lymph node; Macro, macrometastases; Micro, micrometastases ; Pat, patients; SLN, sentinel lymph node.
